# Dental and skeletal components of Class II open bite treatment with a modified Thurow appliance

**DOI:** 10.1590/2176-9451.19.1.019-025.oar

**Published:** 2014

**Authors:** Helder Baldi Jacob, Ary dos Santos-Pinto, Peter H. Buschang

**Affiliations:** 1 Post doc student in Orthodontics, Texas A&M Baylor College of Dentistry.; 2 Full professor in Orthodontics, School of Dentistry - State University of São Paulo/Araraquara.; 3 Professor, Department of Orthodontics, Texas A&M Baylor College of Dentistry.

**Keywords:** Angle Class II malocclusion, Open bite, Orthopedics

## Abstract

**Introduction:**

Due to the lack of studies that distinguish between dentoalveolar and basal changes caused by the Thurow appliance, this clinical study, carried out by the School of Dentistry - State University of São Paulo/Araraquara, aimed at assessing the dental and skeletal changes induced by modified Thurow appliance.

**Methods:**

The sample included an experimental group comprising 13 subjects aged between 7 and 10 years old, with Class II malocclusion and anterior open bite, and a control group comprising 22 subjects similar in age, sex and mandibular plane angle. Maxillary/mandibular, horizontal/vertical, dental/skeletal movements (ANS, PNS, U1, U6, Co, Go, Pog, L1, L6) were assessed, based on 14 landmarks, 8 angles (S-N-ANS, SNA, PPA, S-N-Pog, SNB, MPA, PP/MPA, ANB) and 3 linear measures (N-Me, ANS-Me, S-Go).

**Results:**

Treatment caused significantly greater angle decrease between the palatal and the mandibular plane of the experimental group, primarily due to an increase in the palatal plane angle. ANB, SNA and S-N-ANS angles significantly decreased more in patients from the experimental group. PNS was superiorly remodeled. Lower face height (ANS-Me) decreased in the experimental group and increased in the control group.

**Conclusions:**

The modified Thurow appliance controlled vertical and horizontal displacements of the maxilla, rotated the maxilla and improved open bite malocclusion, decreasing lower facial height.

## Abstract (pt)

**Introdução:**

devido ao número reduzido de estudos que distinguem entre as mudanças dentoalveolares e ósseas produzidas pelo aparelho de Thurow, esse estudo clínico, conduzido pelo departamento de Ortodontia da Faculdade de Odontologia de Araraquara, foi proposto para avaliar as mudanças dentárias e esqueléticas induzidas pelo aparelho de Thurow modificado.

**Métodos:**

a amostra incluiu um grupo experimental de 13 indivíduos, entre 7 e 10 anos de idade, com má oclusão Classe II e mordida aberta anterior, e um grupo controle de 22 indivíduos, com idades, plano mandibular e má oclusão similares. Com base em 14 pontos cefalométricos, 8 ângulos (S-N-ANS, SNA, PPA, S-N-Pog, SNB, MPA, PP/MPA, ANB) e 3 medidas lineares (N-Me, ANS-Me, S-Go), foram avaliados movimentos maxilares e mandibulares horizontais e verticais dentários e esqueléticos (ANS, PNS, U1, U6, Co, Go, Pog, L1, L6).

**Resultados:**

o tratamento produziu diminuição significativamente maior no ângulo entre o plano palatino e o plano mandibular do grupo controle, devido ao aumento do ângulo do plano palatino. Os ângulos A-N-B, S-N-A e S-N-ANS diminuíram significativamente mais no grupo tratado. A PNS sofreu remodelação superior. A altura facial inferior (ANS-Me) diminuiu no grupo tratado, ao passo que aumentou no grupo controle.

**Conclusões:**

o aparelho controlou deslocamento vertical e horizontal da maxila, rotacionou a maxila para melhorar a mordida aberta e diminuiu a altura facial inferior.

## INTRODUCTION

Class II malocclusion can be due to skeletal or dental maxillary protrusion, mandibular retrusion or a combination of factors.^1,2,3^ While Class II malocclusion can be addressed in a number of different ways (i.e. dentoalveolar changes, orthopedic forces to inhibit maxillary growth or stimulate mandibular growth, or surgical repositioning of the mandible in non-growing patients), maxillary protrusion is usually treated with orthopedic forces produced by headgear appliances.^3,4,5^ Headgear appliances can be inserted into bands bonded onto the upper molars or into removable appliances. The issue of whether or not headgear therapy causes skeletal maxillary changes in humans remains controversial.^6-9^

When associated with hyperdivergence and anterior open bite, Class II malocclusions have proven to be a daunting challenge for orthodontists. The position of the tongue as well as thumb and finger sucking are perhaps the best known physical factors that cause open bite malocclusions.^10^ Hyperdivergent open bite subjects have anterior and posterior dentoalveolar heights that tend to be excessive, palatal plane angles that are flatter, as well as increased mandibular plane and gonial angles.^11^ To treat such malocclusion in growing patients, it is necessary to limit maxillary displacement and intrude the molars in order to rotate the mandible upwards and forward.^12,13^

The Thurow appliance was developed to apply distal and vertical forces while controlling molar rotation and tipping produced by forces directed through buccal molar tubes. The original appliance, which incorporates a high-pull headgear and a maxillary acrylic splint that serves as a bite block, has been shown to restrain maxillary growth, distally tip and displace the maxillary teeth, as well as restrain the eruption of posterior maxillary teeth.^14,15^ Because the splint precisely covers the entire maxillary dentition, higher force levels dissipating over a larger surface area can be used. The acrylic smooth surface disoccludes the teeth and effectively eliminates occlusal interferences during force application, which facilitates maxillary tooth movement and allows the mandible to grow unimpeded by the maxilla. The Thurow appliance is thought to be particularly well suited for Class II patients with maxillary prognathism, steep mandibular plane angles and open bites.^16^

It has been reported that the Thurow appliance can be used to decrease the ANB angle, inhibit maxillary horizontal growth, control vertical growth of the maxilla, maintain the mandibular plane angle, move the upper first molars distally, and improve lip relationships.^12,13,16-19^ However, these claims have been based on case reports which have not been compared to control groups. Existing case-control studies were not able to distinguish between dentoalveolar and basal changes produced by the appliance because mandibular and, especially, maxillary superimpositions were not performed.^14,20,21^

The ability to distinguish between skeletal/dental contributions and correction is important not only to ensure that treatment objectives were met, but also to further improve therapies performed with the appliance. Clinically, understanding the effects of Thurow high-pull headgear is vital to understand Class II correction in growing hyperdivergent patients. The aim of this retrospective study was to assess dental and skeletal changes produced by a Thurow high-pull headgear appliance for hyperdivergent patients with open bite and Class II division 1 malocclusion, by means of cephalometric radiographs.

## MATERIAL AND METHODS

### Sample

Fifteen children participated in this retrospective clinical study as a treated group. Recruitment was conducted at the orthodontic clinic of the School of Dentistry - State University of São Paulo/Araraquara. During treatment, two patients moved away from the city.

**Figure 1:**
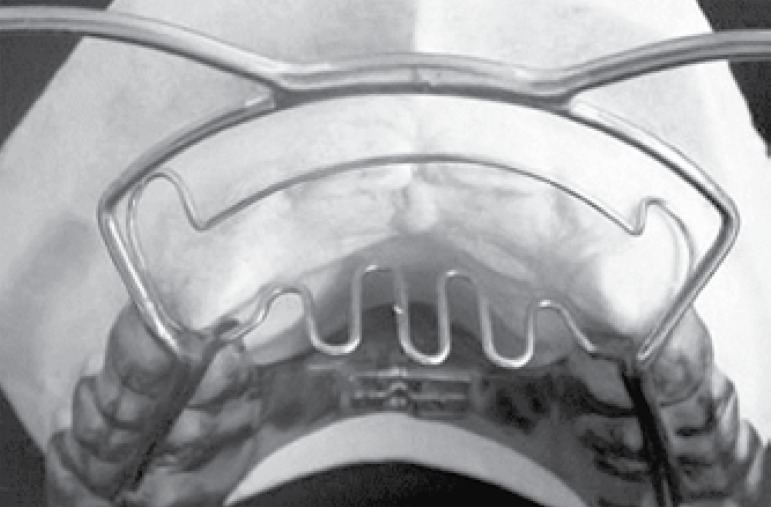
Modified Thurow appliance.

The final treated group included 13 children (1 male and 12 female) with Class II division 1 malocclusion as well as open bite. The children aged between 7 and 10 years old and were treated for 12 months before growth spurt (Table 1). The maxillary splint high-pull headgear comprised an acrylic plate, a vestibular arch, an extraoral arch fixed to the acrylic, a palatal crib, and an expansion screw at the level of the second deciduous molars (Fig 1); and it was based on the appliance introduced by Thurow^16^ and modified by Santos-Pinto.^18^ The acrylic plate extended laterally and occlusally, covering the cusps and approximately one-third of the molars buccal surfaces. Should expansion be necessary, the screw was activated once a week (0.25 mm) for as long as it was needed. The outer bow of the extraoral arch was adjusted so that the line of force of the elastics slightly passed anteroposteriorly through the first and second deciduous molars and between the lower margin of the orbitale, and vertically through the apex of the first molar, which is slightly posterior to the maxilla center of resistance.^22,23^ The high-pull headgear delivered approximately 400 g of force on each side and was worn 14 hours a day. After correction was achieved, the patients wore the headgear for 8 to 10 hours during sleep. They were seen monthly so that the splint could be adjusted, if necessary.

**Table 1:** Pre-treatment and follow-up ages of the treated (Thurow appliance) and untreated (control) groups.

Group		Sample size	Mean ± SD	Prob.
Initial	Treated	13	8.85 ± 0.73	0.912
	Untreated	22	8.82 ± 0.73	
Final	Treated	13	9.84 ± 0.70	0.933
	Untreated	22	9.82 ± 0.73	

The untreated control group included children who were followed longitudinally at the Human Growth Research Center, University of Montreal, Canada. They were from three different school districts in Montreal and represented various socioeconomic strata.^24^ The control group sample comprised 22 patients (2 males and 20 females) with Class II division 1 who were at the same age, with the same sex and mandibular plane angle when compared to the treated sample.

### Cephalometric methods

**Figure 2:**
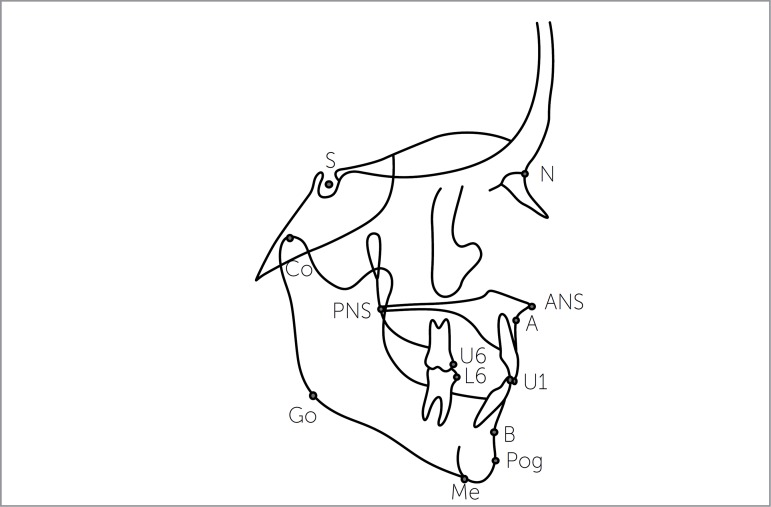
Cephalometric landmarks digitized; (S) sella, (N) nasion, (PNS) posterior nasal spine, (ANS) anterior nasal spine, (A) A-point, (Co) condylion, (Go) gonion, (Me) menton, (Pog) pogonion, (B) B-point, (U6) maxillary mesial molar, (U1) maxillary incisor tip, (L6) mandibular mesial molar, (L1) mandibular incisor tip.

Lateral cephalograms were obtained at the beginning of the treatment (T_1_) and at the follow-up appointment (T_2_) in the treated group. In the control group, the lateral cephalograms were obtained after one year, at least fifteen days before or after the initial day. The cephalograms were taken with the head positioned according to the Frankfort horizontal plane, and the lateral cephalometric tracings were performed on acetate paper. The tracings were digitized and analyzed with Viewbox 3.1-Cephalometric Software (Dhal Software, Athens, Greece) by one investigator. The linear measurements were adjusted to eliminate magnification. The analyses described growth and treatment changes of fourteen skeletal landmarks (Fig 2).

**Figure 3:**
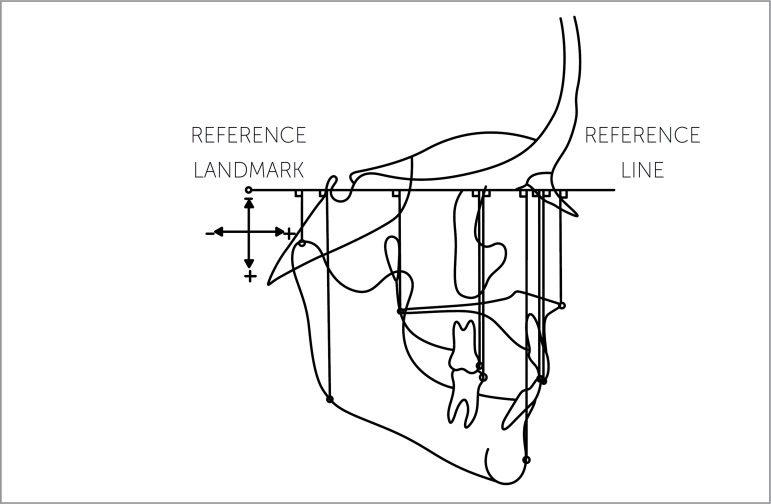
Horizontal and vertical cephalometric landmarks position measured parallel and perpendicular to the reference line (SN = -7°).

The horizontal and vertical movements of the selected landmarks were described on the basis of a horizontal reference line (RL), which was oriented in T_1_ based on the sella-nasion plane with -7 degrees. For example, the horizontal change in the position of pogonion was measured parallel to the RL (distance between the pogonion projection to a reference point fixed 100 mm behind the sella), while the vertical change was measured perpendicular to the RL (Fig 3). In general, tooth movements were calculated based on tracings superimposed to the stable cranial base structures, as described by Björk and Skieller.^25^ To determine the actual movement of incisors and molars, maxillary and mandibular superimpositions were also performed, as described by Björk and Skieller.^25,26^ After partial superimposition, tooth movements were subtracted from the overall tooth movements in order to estimate the movement of the skeletal bases. Horizontally, an anterior change was recorded as positive, whereas a posterior change was recorded as negative. Vertically, a superior change was recorded as negative, whereas an inferior change was recorded as positive (Fig 3).

Replicate analysis of 26 subjects showed small, but statistically significant systematic errors for the ANS horizontal (0.31 mm) and Go vertical (-0.21 mm). Random method errors ranged from 0.15 to 0.46 mm with PNS horizontal showing the largest random error.^27^

### Statistical methods

The measurements were transferred to SPSS software (version 15.0, SPSS, Chicago, USA) for evaluation. Based on Skewness and Kurtosis, the variables were normally distributed. T-tests were used to compare the groups. A probability level of 0.05 was used to determine statistical significance.

## RESULTS

T-tests showed significant (P < 0.05) differences between groups prior to treatment of five out of the 11 traditional variables measured (Table 2). In comparison to the control group, the treated group initially had greater ANB angles, smaller palatal plane angles and greater anterior and posterior facial heights. Analysis of covariance demonstrated that none of the traditional pretreatment variables were related to post-treatment changes.

**Table 2:** Comparison of pretreatment values between treated and untreated groups.

Variable		Treated		Untreated		Prob.(difference)
		Mean ± SD		Mean ± SD		
S-N-ANS	Deg	87.45	5.88	86.38	2.62	0.548
SNA	Deg	82.37	5.93	81.27	3.19	0.550
PPA	Deg	3.99	3.40	6.91	2.79	**0.016**
SNPog	Deg	77.25	5.14	77.28	3.02	0.981
SNB	Deg	77.21	5.45	77.50	3.10	0.862
MPA	Deg	35.97	5.30	36.27	3.60	0.855
PP/MPA	Deg	31.96	4.62	29.19	3.41	0.076
ANB	Deg	5.16	1.90	3.72	2.00	**0.046**
N-Me	mm	96.58	4.58	92.03	4.02	**0.007**
ANS-Me	mm	58.23	3.99	53.02	2.66	**0.001**
S-Go	mm	60.82	5.25	57.27	3.90	**0.047**

Regardless of pretreatment measures, the treatment yielded significant differences. The palatal plane angle increased in the treated group and remained unchanged in the control. This difference, along with the greater, although not statistically significant decrease in the MPA, resulted in a significantly greater decrease in the PP/MPA of the treated group. The ANB angle significantly decreased more in treated patients than in the control group, primarily due to a significant treatment decrease in the SNA angle. While lower face height increased significantly in the control, it significantly decreased in the treated group.

In comparison to the treated group, which showed no statistically significant horizontal displacement, the maxilla and maxillary teeth of the control group were anteriorly displaced approximately 0.7 mm over the observation period (Table 4). Although the treated group showed anterior displacement of the mandible, the changes were not statistically significant. All mandibular landmarks of the control group showed significant anterior displacement, except for the condylion. None of the differences regarding horizontal displacement between groups were statistically significant.

**Table 3:** Comparison of changes between treated and untreated groups.

Variable		Treated		Untreated		Prob.(difference)
		Mean ± SD		Mean ± SD		
S-N-ANS	Deg	-2.75	1.20	0.08	1.45	**<0.001**
SNA	Deg	-0.94	0.80	0.03	1.15	**0.007**
PPA	Deg	2.14	1.59	0.07	0.85	**<0.001**
S-N-Pog	Deg	0.27	1.12	0.33	0.66	0.871
SNB	Deg	0.16	0.95	0.22	0.59	0.846
MPA	Deg	-0.61	1.63	-0.17	0.99	0.392
PP/MPA	Deg	-2.73	1.92	-0.23	1.12	**0.001**
A-N-B	Deg	-1.10	0.88	-0.12	1.15	**0.010**
N-Me	mm	1.64	1.55	2.36	1.52	0.198
ANS-Me	mm	-0.92	1.44	1.14	1.26	**<0.001**
S-Go	mm	1.68	1.68	1.64	0.89	0.938

**Table 4:** Horizontal skeletal and dental changes in treated and untreated patients (positive value = forward direction; negative value = backward direction).

Horizontal values			
Displacement			
Variable	Treated	Untreated	Prob. (difference)
	Mean ± SD	Mean ± SD	
ANS	0.01 ± 0.83	**0.72** ± 1.16	0.060
PNS	0.02 ± 0.84	**0.74** ± 1.21	0.068
U1	0.15 ± 1.62	**0.71** ± 1.56	0.315
U6	0.12 ± 1.35	**0.72** ± 1.41	0.229
Co	-0.42 ± 2.42	0.15 ± 1.73	0.418
Go	0.63 ± 2.12	**0.93** ± 1.37	0.618
Pog	1.32 ± 2.66	**1.44** ± 1.84	0.878
L1	0.59 ± 2.17	**0.86** ± 1.38	0.654
L6	0.55 ± 2.11	**0.86** ± 1.37	0.592
Remodeling / tooth movement			
Variable	Treated	Untreated	Prob. (difference)
	Mean ± SD	Mean ± SD	
ANS	0.35 ± 1.18	**0.78** ± 1.36	0.428
PNS	-1.14 ± 1.93	**-0.81** ± 1.14	0.699
U1	-0.02 ± 1.53	**0.80** ± 1.14	0.091
U6	0.33 ± 1.15	**0.56** ± 1.16	0.625
Co	0.25 ± 2.54	-0.54 ± 1.71	0.159
Go	-0.55 ± 1.64	**-1.45** ± 1.31	0.053
Pog	0.02 ± 0.13	-0.07 ± 0.25	0.271
L1	0.74 ± 1.44	0.34 ± 0.94	0.203
L6	**0.98** ± 1.28	0.36 ± 0.96	0.121

Bold = significant change between initial and final radiographs.

Based on the maxillary superimpositions, the treated group demonstrated no statistically significant difference with regard to horizontal remodeling or tooth migration; the control group showed anterior and posterior remodeling of ANS and PNS, respectively, and mesial drift of the incisors molars. Except for the gonion of the control group, which drifted posteriorly, and for the lower molar of the treated group, which moved mesially, none of the mandibular measures showed statistically significant horizontal changes. While several of the group comparisons were at a significant level, none of the differences were statistically significant.

Both groups showed statistically significant inferior displacement, with no significant differences between groups (Table 5). The maxilla was inferiorly displaced for approximately 1 mm. The posterior and anterior aspects of the mandible were inferiorly displaced for approximately 2.9 to 3.4 mm and 1.5 to 2.3 mm, respectively. While ANS showed no significant remodeling changes, PNS showed slight superior drift in the treated group and inferior drift in the control group, with statistically significant differences. The maxillary molars of the treated group showed no vertical changes, whereas the control molars erupted approximately 0.8 mm. There was little or no group difference in mandibular remodeling and tooth movements. Condylion showed the greatest growth (2.6 to 2.8 mm), gonion drifted superiorly, the incisors erupted 0.8 to 1.2 mm and the mandibular molars erupted 0.8 to 0.9 mm.

**Table 5:** Vertical skeletal and dental changes in treated and untreated patients (positive value = inferior direction; negative value = superior direction).

Vertical values			
Displacement			
Variable	Treated	Untreated	Prob.(difference)
	Mean ± SD	Mean ± SD	
ANS	**0.80** ± 1.47	**0.98** ± 1.54	0.740
PNS	**1.02** ± 1.08	**1.00** ± 0.69	0.964
U1	**0.83** ± 1.42	**1.00** ± 1.48	0.749
U6	**0.96** ± 0.88	**0.96** ± 0.89	0.995
Co	**2.94** ± 2.30	**3.53** ± 2.13	0.448
Go	**2.86** ± 2.25	**3.40** ± 1.95	0.466
Pog	**1.51** ± 1.82	**2.31** ± 1.28	0.180
L1	**1.37** ± 1.97	**2.23** ± 1.36	0.181
L6	**1.89** ± 1.70	**2.59** ± 1.22	0.168
Remodeling / eruption			
Variable	Treated	Untreated	Prob.(difference)
	Mean ± SD	Mean ± SD	
ANS	0.22 ± 0.44	0.37 ± 1.75	0.775
PNS	**-0.50** ± 0.57	**0.21** ± 0.43	**0.001**
U1	**1.03** ± 0.91	**0.93** ± 1.45	0.243
U6	0.33 ± 1.15	**0.82** ± 0.95	0.073
Co	- **2.63** ± 2.69	**-2.82** ± 1.75	0.371
Go	**-1.25** ± 1.69	**-1.74** ± 1.85	0.489
Pog	**0.11** ± 0.14	0.04 ± 0.71	0.699
L1	**-1.24** ± 1.49	**-0.82** ± 1.14	0.319
L6	**-0.94** ± 0.86	**-0.84** ± 0.77	0.448

Bold = significant change between initial and final radiographs.

## DISCUSSION

The modified Thurow appliance clearly restricted the forward growth of the maxilla. The treated subjects showed a decrease of 2.8^o^ and 0.9^o^ in S-N-ANS and SNA, respectively. The angles decreased in the treated group because the maxilla maintained its anteroposterior position while the nasion continued to drift anteriorly. The control group showed little or no change in SNA or S-N-ANS because the maxilla moved forward along with the nasion. This distinction is important because previous studies have reported, based solely on decreases in SNA or S-N-ANS, that headgears used to correct Class II malocclusions are generally effective in posteriorly redirecting maxillary growth.^14,16,20,21,28-31^

Most studies have not assessed the actual anteroposterior movement of the maxilla. Baumrind et al,^8^ who used the biologically defined "best fit" of palatal structures, showed small, but definite posterior movement of ANS. In the present study, ANS was not displaced posteriorly, perhaps due to the more superiorly oriented forces produced by the high-pull headgear.

The modified Thurow appliance produced 2.1^o^ posteriorly, or a backward rotation of the palatal plane. In contrast with the control group, the treated group showed no statistically significant changes of the palatal plane angle, as expected for untreated subjects over a similar time period.^33-36^ Other studies evaluating the effects of high-pull forces have all shown backward rotation of the palatal plane.^14,20,30,37,38^

In some situations, the orthodontist wants to prevent maxillary rotation, in which case the high-pull forces should be directed through the maxilla center of resistance.

In this study, the headgear forces were purposely directed behind the dental and maxillary centers of resistance in order to help correcting the open bite. Rotation of the palatal plane also explains the decrease observed in the lower anterior face height of the treated group.^20,31^ Lower anterior face height of the control group increased, as expected during growth of untreated subjects.^33,34,36^

The modified Thurow appliance used in the present study had no real treatment effects on the anteroposterior mandibular position. The S-N-Pog and SNB angles did not significantly change in either treated or control group. Previous studies also show no changes in the anteroposterior position of the mandible.^14,20,21,32,39^ Lahaye et al,^40^ who evaluated methods commonly used to correct Class II skeletal malocclusions, including headgears and Herbst appliances, found no appreciable significant improvements in anteroposterior chin position. The authors stated that skeletal Class II correction in growing adolescents results primarily from maxillary growth restriction or inhibition.

The mandibular plane angle did not show statistically significant differences between groups either. Both groups showed forward rotation during the observation period. Most previous studies have shown that the mandibular plane angle changed or was maintained during treatment.^14,20,21,32,41^ Except for Bhatia and Leighton,^36^ who reported a slight increase for males and stable relations for females, previous longitudinal studies of untreated children have also shown decreases in the MPA between 10-15 years, ranging from 0.8 to 3.5^o^.^33,34,35^

## CONCLUSION

1. The modified Thurow appliance held the maxilla and caused a slight backward rotation of the palatal plane.
2. The maxillary molars of the treated group showed neither horizontal nor vertical changes. The upper incisors were retroclined, but no significant change was observed over time.
3. Except for the lower molars, which moved mesially in the treated group, no treatment effect was observed in the mandible.
4. Lower facial height decreased in the treated group.
